# A novel Ecotin-Ubiquitin-Tag (ECUT) for efficient, soluble peptide production in the periplasm of *Escherichia coli*

**DOI:** 10.1186/1475-2859-8-7

**Published:** 2009-01-21

**Authors:** Michael Paal, Thomas Heel, Rainer Schneider, Bernhard Auer

**Affiliations:** 1 Austrian Center of Biopharmaceutical Technology, Muthgasse 18, A-1190 Vienna, Austria; 2 Institute of Biochemistry, University of Innsbruck, Peter-Mayr-Strasse 1a, A-6020 Innsbruck, Austria

## Abstract

**Background:**

Many protocols for recombinant production of peptides and proteins include secretion into the periplasmic space of *Escherichia coli*, as they may not properly fold in the cytoplasm. If a signal peptide is not sufficient for translocation, a larger secretion moiety can instead be fused to the gene of interest. However, due to the covalent linkage of the proteins, a protease recognition site needs to be introduced in between, altering the N-terminus of the product. In the current study, we combined the ubiquitin fusion technology, which allows production of authentic peptides and proteins, with secretion by the perpiplasmic protease inhibitor ecotin.

**Results:**

Different fusion constructs, composed of *ecotin*, *mouse ubiquitin b *and a model peptide, were expressed in *E. coli *BL21(DE3). The fusion proteins were translocated into the periplasmic space and the ecotin signal peptide was cleaved off. Under the control of the lacUV5 promoter at 24°C we obtained 18 mg periplasmic recombinant protein per gram dry cell weight. However, vigorous expression with the T7 promoter caused outer membrane permeabilization and leakage of the fusion protein into the culture medium. Target peptides were released from hybrid proteins by the deubiquitinating enzyme ubiquitin c-terminal hydrolase-L3 *in vitro*. MALDI TOF-TOF mass spectroscopy confirmed accurate cleavage.

**Conclusion:**

This newly described method represents a useful technique for the production of authentic soluble peptides in the periplasm of *E. coli*. In addition, larger proteins might also be produced with the current system by the use of ubiquitin specific proteases, which can cleave off larger C-terminal extensions.

## Background

The gram-negative bacterium *E. coli *is widely used as host for the production of recombinant proteins, due to well-known genetics, simple handling and inexpensive fast high-density cultivations [1,2]. Recombinant expressed proteins in *E. coli *can be directed to the cytoplasm, inner or outer membrane, periplasm or the growth medium.

Cytoplasmatic expression is desired because of high yields of soluble product [3], but recombinant protein may accumulate in inclusion bodies (IB). Although biologic activity must be restored by *in vitro *renaturation procedures [4], IB can be valuable for heterologous gene expression [5]. One strategy to prevent IB formation involves fusion of the gene of interest downstream of the small eukaryotic protein ubiquitin [6]. The ubiquitin moiety enhances fusion protein yield and increases solubility due to intrinsic chaperonin properties [7]. This fusion system also allows production of proteins and peptides with an authentic N-terminus by the use of deubiquitinating enzymes (DUB), cleaving the amide bond at the C-terminus of ubiquitin [8]. As *E. coli *lacks the ubiquitin system, release of target products can be conducted by protease addition [9,10].

Protein export out of the cytoplasm has several advantages compared to intracellular production, due to 1) better folding (especially if disulfide-bond formation is required), 2) simplified downstream processing, 3) higher stability, 4) and correct N-terminal processing [11,12]. Translocation of target proteins across the inner membrane requires a signal peptide. However, presence of a signal sequence alone does not ensure secretion into the periplasmic space [13,14]. Thus, a larger secretion moiety can be linked to the target gene. Recently, a secretion system was described, using the periplasmic protease inhibitor ecotin [15] as fusion partner for the production of native pepsinogen and proinsulin in *E. coli *[16,17].

In the present work we established a new expression system, combining periplasmic secretion and the ubiquitin fusion technology. We secreted different fusion proteins, consisting of *ecotin, mouse ubiquitin b *and a target peptide. A deubiquitinating enzyme precisely cleaved off peptides from recombinant fusion proteins *in vitro*.

## Methods

Water used in all experiments was Milli-Q ultrapure water (Millipore purification system). Transformed *E. coli *cells were cultivated in TY growth medium supplemented with 50 μg/ml kanamycin. For recombinant plasmid isolation *E. coli K12 *DH5α strain was used, whereas protein expression was performed in BL21(DE3).

Restriction enzymes, GoTaq^® ^DNA polymerase, including the PCR buffer, were from Promega. Molecular mass standard used for SDS-PAGE, rapid DNA ligation kit, *Pfu *DNA polymerase and 10 × MgSO_4_-PCR buffer were obtained from Fermentas. Tris-Glycine gels were purchased from Invitrogen. Protran BA 83 nitrocellulose membrane was obtained from Whatman, polyvinylidene difluoride (PVDF) membrane from Hybond. mouse anti-GroEL monoclonal antibody was purchased from Stressgen Bioreagents, goat anti-Mouse IgG (HRP conjugated) from Invitrogen, anti-Maltose Binding Protein (MBP) monoclonal antibody (HRP conjugated) from New England Biolabs. Syringe filters (pore size 0.45 μm) were from Sartorius, 10 kDa molecular weight cut-off ultrafiltration devices (Centriprep Ultracel YM-10 tubes, series 8000 stirred cell including Ultracel YM-10 membranes) from Millipore. Gravity flow Strep-Tactin^® ^Sepharose^® ^column and Strep-Tactin^® ^HRP conjugate were obtained from IBA. The BCA™ protein assay kit was obtained from Pierce. Rabbit ubiquitin C-terminal hydrolase-L3 (UCH-L3) was from Sigma.

### Construction of expression plasmids

A Strep-Tag II octapeptide (abbreviated Strep) was fused C-terminal to each construct, allowing immunodetection.

The *ecotin *gene [GenBank: M60876] was amplified from the *E. coli *K12 DH5α strain with GoTaq^® ^DNA polymerase, using the primers NdeI Ecotin F and Ecotin-Strep SalI R (Table 1). The amplified fragment was ligated into NdeI and SalI digested pET30a vector, resulting in pET30a Ecotin-Strep (Table 2).

**Table 1:** Oligonucleotides used in this study

**Primers^a^**	**Sequences (5'-3')**
NdeI Ecotin F	GATATACATATGAAGACCATTCTACCTGCAGTA
Ecotin-Strep SalI R	TATAGTCGACTTATTTTTCGAACTGCGGGTGGCTCCAGCGAACTACCGCGTTGTCAAT
Ecotin-Ubi (AS2–7) R	CAGGGTCTTCACGAAGATCTGGCGAACTACCGCGTTGTCAAT
Ubi (AS2–7) F	CAGATCTTCGTGAAGACCCTGACC
Ubi-Strep XhoI R	TATACTCGAGTTATTTTTCGAACTGCGGGTGGCTCCAGCCACCCCTCAGACGGAGGAC
lacUV5p SphI F	CCCAGGCTTTACACTTTATGCTTCCGGCTCGTATAATGTGTGGAATTGTGAGCGGATAACAATTT
lacUV5p XbaI R	CTAGAAATTGTTATCCGCTCACAATTCCACACATTATACGAGCCGGAAGCATAAAGTGTAAAGCCTGGGCATG
Ubi-FLS XhoI 1 F	TATGAGTCGACCCTGCACCTGGTCCTCCGTCTCCGCGGTG
Ubi-FLS XhoI 2 R	CCGCCACCTTTATCGTCATCATCTTTATAGTCACCACCGCGGAGACGGAG
Ubi-FLS XhoI 3 F	ATGACGATAAAGGTGGCGGAGGTAGCGGTGGAGGAGGCAGCTGGAGCCAT
Ubi-FLS XhoI 4 R	TATACTCGAGTTATTTTTCAAACTGCGGATGGCTCCAGCTGCCTC

^a ^The oligonucleotides contain the letters: F – forward or R – reverse

**Table 2:** Plasmids and corresponding expression products

**Vectors^a^**	**Gene cloned**	**Resulting vectors^a^**	**Expression product**
pET30a	Ecotin-Strep	pET30a Ecotin-Strep	Ecotin-Strep
pET30a	Ecotin-Ubiquitin-Strep	pET30a Eco-Ubi-Strep	Ecotin-Ubiquitin-Strep
pET30a lacUV5p	Ecotin-Ubiquitin-Strep	pET30a lacUV5p Ecotin-Ubiquitin-Strep	Ecotin-Ubiquitin-Strep
pET30a lacUV5p Ecotin-Ubiquitin-Strep	FLS	pET30a lacUV5p Ecotin-Ubiquitin-FLS	Ecotin-Ubiquitin-FLS

^a ^plasmids with the lacUV5 promoter are named pET30a lacUV5p

A three-step gene assembly with *Pfu *DNA Polymerase generated the *Ecotin-Ubiquitin-Strep *fusion. First, *ecotin *with an ubiquitin extension was amplified from vector pET30a Ecotin-Strep with the primers NdeI Ecotin F and Ecotin-Ubi (AS2–7) R. Next, *ubiquitin *[GenBank: NM_011664] was obtained from another plasmid with the Ubi (AS2–7) F and Ubi-Strep XhoI R oligonucleotides. Both genes were linked and amplified in a follow up PCR with the primers NdeI Ecotin F and Ubi-Strep XhoI R. The resulting fragment was cloned into NdeI and XhoI digested pET30a vector.

The pET30a T7 promoter (T7p) was exchanged in different vectors with the artificial lacUV5 promoter (lacUV5p). Two oligonucleotides with 65 complementary bases, lacUV5p SphI F and lacUV5p XbaI R, were directly ligated into SphI and XbaI digested pET30a plasmids.

**Figure 1:**
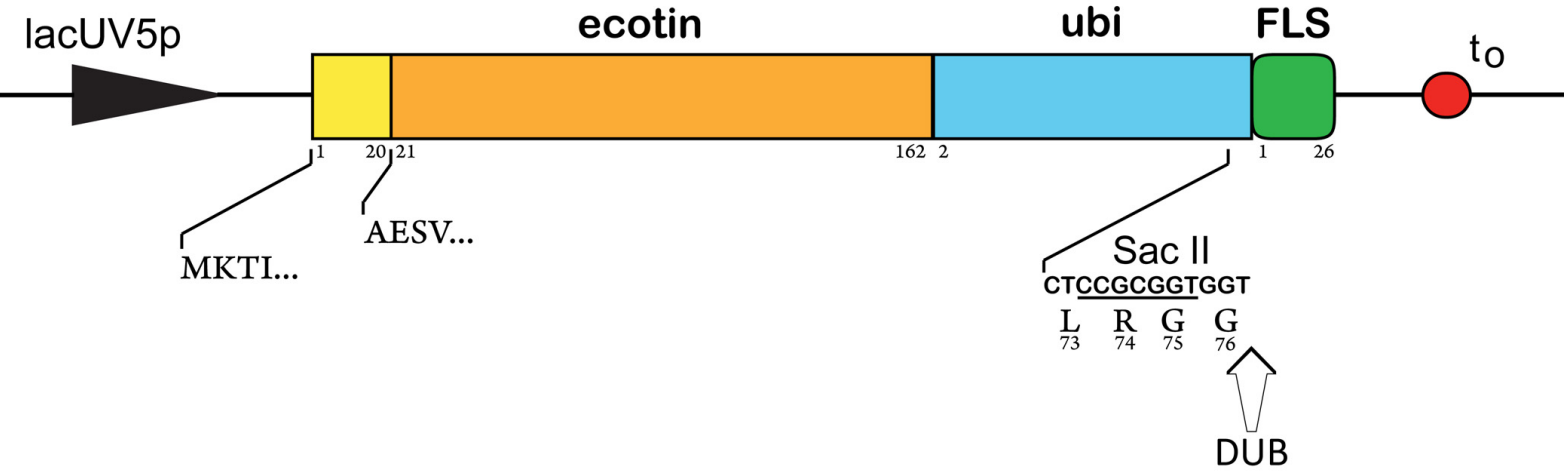
**Schematic representation of the vector used for Ecotin-Ubiquitin-FLS expression**. Amino acids of the corresponding genes are numbered. Full-length *ecotin *with the intrinsic signal sequence (1–20) was cloned upstream *of mouse ubiquitin B *(ubi, 2–76), followed by the *FLS *(1–26) target peptide. The cleavage site for a deubiquitinating enzyme (DUB) is indicated with an arrow. A SacII cleavage was introduced at the *ubiquitin *C-terminus by PCR. t_0_, transcription termination region.

The SalI cleavage site within the *ubiquitin *gene was used to couple the new target peptide *FLS *to its C-terminus. The peptide coding sequence, including a c-terminal part of *ubiquitin*, was generated with a two-step gene synthesis protocol using *Pfu *DNA polymerase. Complementary Ubi-FLS XhoI primers were annealed and extended during a first PCR procedure. A second PCR run with the primers Ubi-FLS XhoI 1 F and Ubi-FLS XhoI 4 R generated the full-length product. Insertion into SalI and XhoI cleaved pET30a lacUV5p *Ecotin-Ubiquitin-Strep *led to formation of the new fusion gene *Ecotin-Ubiquitin-FLS *(Fig. 1).

### Small scale fermentation

Expression plasmids were transformed in *E. coli *BL21(DE3) and overnight cultures inoculated at 37°C and 225 rpm. The cultures were diluted 1:15 with fresh medium and grown to an OD_600 _of 0.5–0.7 at 37°C. Recombinant protein synthesis was induced adding 1 mM IPTG. Expression with T7p was carried out for 3 h at 37°. In contrast cells carrying pET30 lacUV5p plasmids were incubated at 24°C for 3 to 5 h.

### Cell fractionation

Isolation of the periplasm was performed at 24°C with a gentle osmotic shock procedure to minimize *E. coli *cell disruption during preparation (modified from [18]). After expression, a given culture volume (OD_600 _between 1.5 and 3.0) was centrifuged at 3000 g for 10 min and the supernatant filtrated through a 0.45 μm membrane. The pellet was completely suspended in one culture volume of osmolysis buffer (100 mM TRIS-HCl pH 7.8, 15.4% sucrose, 3 mM EDTA) and incubated at 50 rpm for 10 min, followed by centrifugation. The supernatant was discarded and the pellet dissolved in one culture volume water for 10 min at 50 rpm. Afterwards the suspension was centrifuged for 15 min at 3000 g. The supernatant, containing periplasmic proteins, was decanted and filtrated (membrane, 0.45 μm pore size). Subsequently, the spheroblasts were taken up in a culture volume lysis buffer (20 mM Na_2_HPO_4 _pH 8.0, 75 mM NaCl, 5 mM EDTA) and disrupted with a French press (American Instruments Co., Inc). Aliquots of the lysate were collected and centrifuged at 14000 rpm for 15 min. The supernatant contained soluble cytoplasmic protein, whereas the pellet represented the insoluble protein fraction. For SDS-PAGE analysis culture supernant, periplasmic and cytoplasmic fraction samples were precipitated with TCA and all pellets solubilized in loading buffer (62.5 mM Tris-HCl pH 6.8, 10% glycerol, 2% SDS, 0.0025% bromophenol blue, 50 mM DTT).

### Concentration and purification of recombinant protein

For the scaled process, 25 ml 10 × buffer W (1 M Tris-HCl pH 8.0, 1.5 M NaCl, 10 mM EDTA) were mixed with 225 ml filtrated periplasm. Then the suspension was concentrated to 1 ml with ultrafiltration devices. After removal of precipitated proteins by centrifugation, the supernatant was applied on a preequilibrated 1 ml gravity flow Strep-Tactin^® ^Sepharose^® ^column and purified. Recombinant protein was eluted in 6 × 0.5 ml buffer EB (100 mM Tris-HCl pH 8.0, 150 mM NaCl, 1 mM EDTA, 2 mM desthiobiotin). The eluate fractions were pooled and concentrated to 1 ml with a Centriprep YM-10 tube.

### Enzymatic digestion reaction

Enzymatic digestions of fusion proteins were performed *in vitro *at 20°C with 0.05 nmol/ml rabbit UCH-L3 and an enzyme/substrate molar ratio of up to1:2750. Either raw periplasmic extract or Strep-Tactin purified recombinant protein (in buffer EB) were used for cleavage. If extract served as substrate it first had to be adapted to UCH reaction buffer conditions (50 mM Tris-HCl pH 8.5, 1 mM EDTA). To achieve maximal enzyme activity, 10 mM MTG or DTT were added to the digestion solution. Cleavage was stopped by addition of TCA or storage at 4°C.

### Western blots

Cellular integrity after expression and osmotic shock treatment was surveyed by Western blots with antibodies to the periplasmic MBP, and cytoplasmic GroEL marker proteins. Full-length recombinant protein, including Strep-Tag II, was detected with Strep-Tactin^® ^HRP conjugate. Cell fraction samples were separated on 4–20% Tris-Glycine gels and the proteins electrophoretically transferred onto nitrocellulose membranes.

### Protein quantification

Quantitative densitometric analysis of target proteins in comparison to BSA standards on Coomassie stained gels assessed secretion efficiency. Gels were photographed and analyzed with AlphaEaseFC software (Alpha Innotech Corporation). Protein concentration of purified recombinant protein was determined with the BCA™ protein assay.

### N-terminal amino acid sequencing

Recombinant protein (~100 pmol) was blotted onto a PVDF membrane, the Coomassie stained band excised with a razor blade and sequencing performed by automated Edman degradation with a Procise Model 492 micro-sequencer (Applied Biosystems, Norwalk, CT).

### Mass spectrometry

Single and tandem mass spectroscopy was done for purified Ecotin-Ubiquitin-FLS after UCH-L3 digestion (4800 Plus MALDI TOF/TOF™ Analyzer, Applied Biosystems, Norwalk, CT).

## Results and discussion

### Control expression of ecotin

First, we surveyed if Strep-Tag II labeled ecotin could be secreted into the periplasm by conventional expression with the pET system at 37°C. SDS-PAGE analysis of the cellular fractions unveiled two different products (see additional file 1: Ecotin-Strep expression with the pET system). The larger protein resembled unprocessed Ecotin-Strep, where the signal sequence had not been cleaved off by *E. coli *signal peptidase. It was mainly sequestered in IB. The smaller product instead was predominantly directed to the culture supernatant and the signal peptide cleaved off (the sequence AESV after the cleavage site was confirmed by N-terminal amino acid sequencing). As Maltose Binding Protein was detected in the culture supernatant we suggested that outer membrane leakage was responsible for extracellular secretion [19,20].

### Expression of the Ecotin-Ubiquitin-Strep fusion protein

**Figure 2:**
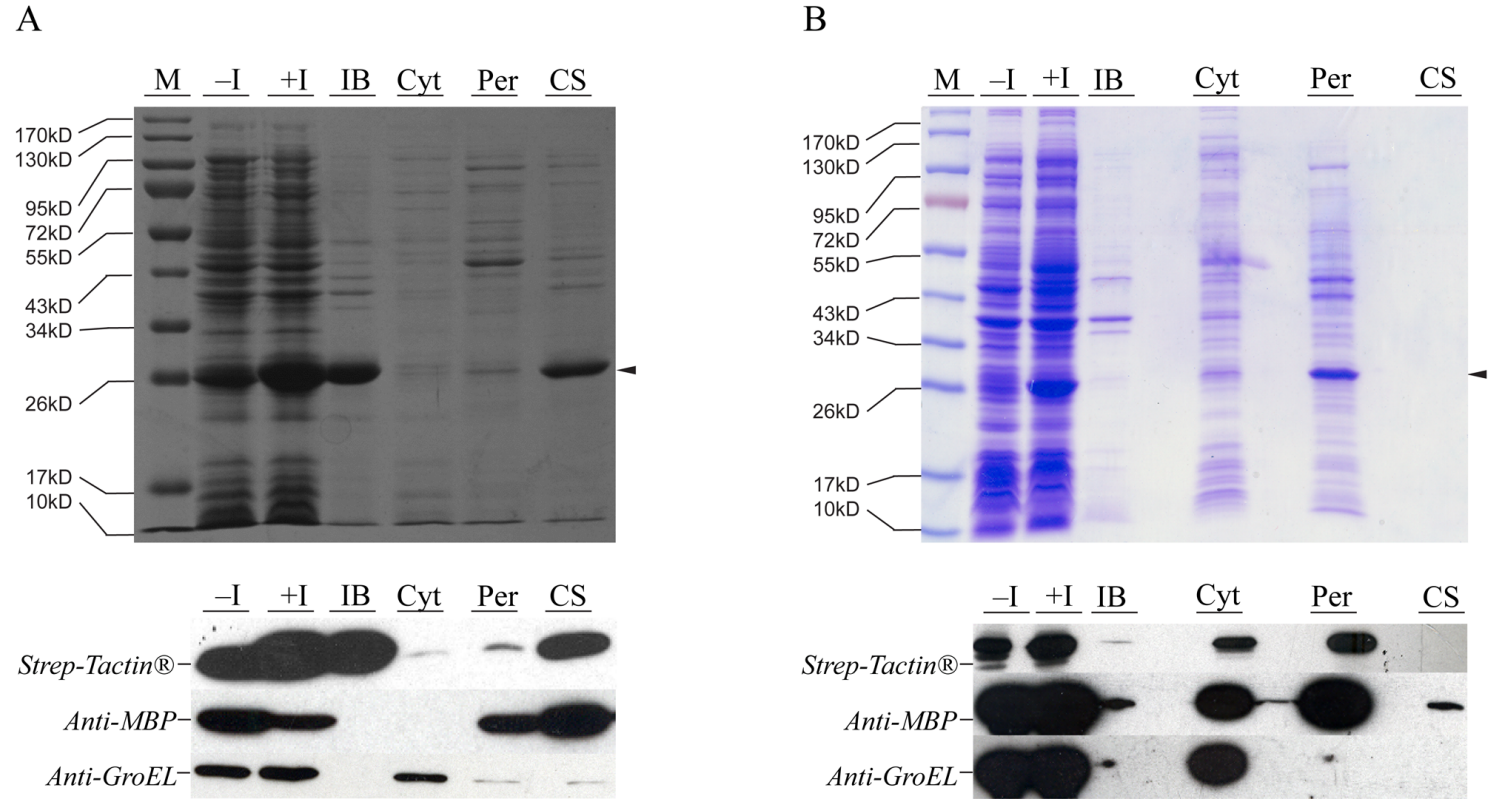
**Cellular localization of Ecotin-Ubiquitin-Strep depending on different expression conditions**. Expression and secretion of fusion proteins was analyzed by SDS-PAGE. Western blots with antibodies to the periplasmic MBP and cytoplasmic GroEL marker proteins assessed proper cell fractionations. A) Cultivation at 37°C with T7 promoter, B) lacUV5p-dependant expression at 24°C. M: protein marker, -I and +I: before and after induction of expression, IB: inclusion bodies, Cyt: cytoplasm, Per: periplasm, CS: culture supernatant. The arrow indicates secreted fusion protein (25.55 kDa).

Since *ecotin *was efficiently expressed and secreted it was used to promote translocation of *ubiquitin*. *Ecotin *and *Ubiquitin-Strep *were fused by PCR, introduced into pET30a and expressed at 37°C. Cell fractionation was surveyed by Western blots using anti-GroEL and-MBP antibodies. Ecotin-Ubiquitin-Strep protein behaved similar to Ecotin-Strep. Processed fusion protein was detected mainly in the culture medium, the uncleaved variant instead aggregated in IB (Fig. 2A).

Inclusion bodies can form when the capacity of the export machinery is overwhelmed by the amount of recombinant protein. Reducing the expression rate can therefore reduce IB formation [21-23]. Thus, we exchanged the T7 promoter (T7p) with the weaker artificial lacUV5 promoter (lacUV5p) and conducted all further expressions at 24°C. Under these conditions nearly the entire recombinant protein was translocated into the periplasm (Fig. 2B) and the signal peptide cleaved off. Outer membrane permeabilization and IB formation were not observed. A concentration of ~18 mg periplasmic fusion protein g^-1 ^dry cell weight was obtained as calculated by quantitative densitometry.

### Cleavage reactions with UCH-L3

Whereas ubiquitin fusion proteins are endogenously processed in yeast, cleavage of recombinant protein in *E. coli *requires either *in vivo *co-expression of a DUB [10] or *in vitro *proteolytic digestion [9]. Since cytoplasmic cleavage of the Ecotin-Ubiquitin-Tag would interfere with secretion of target product, hydrolysis had to be carried out *in vitro*.

**Figure 3:**
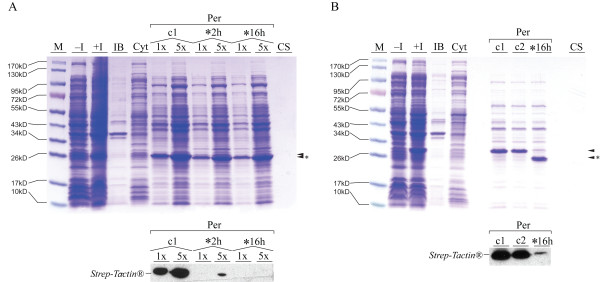
**Release of the (A) Strep and (B) FLS peptides from Ecotin-Ubiquitin fusion proteins by UCH-L3**. Expression and cleavage of the fusion proteins in the periplasmic extract was analyzed by SDS-PAGE and Strep-Tactin^® ^HRP immunoblots. M: protein marker, -I and +I: before and after induction of expression, IB: inclusion bodies, Cyt: cytoplasm, Per: 1 × and 5×, single and fivefold amount of periplasmic protein, c1: periplasmic extract untreated, c2: periplamic extract incubated in UCH buffer, *: UCH-L3 digested periplasm, CS: culture supernatant. The upper arrow indicates intact fusion protein, the lower one with the asterisk the major cleavage moiety Ecotin-Ubiquitin (24.51 kDa).

In our experiments we used Ubiquitin C-terminal hydrolase-L3, which cleaves small peptide extensions with high efficiency and low sequence preference, except those containing proline at the scissile bond [24]. We assessed if the Strep-Tag II peptide with 8 amino acids (aa) in length could be released from periplasmic Ecotin-Ubiquitin-Strep fusion protein by *in vitro *digestion. Crude periplasmic extract of an expression was taken up in UCH reaction buffer including DTT. The solution was subsequently incubated with 0.05 nmol UCH-L3 enzyme per ml reaction volume, corresponding to a 1:9 enzyme/substrate molar ratio. TCA precipitated periplasmic extract served as negative control. Cleavage was efficient and almost complete after 2 h of incubation (Fig. 3A). Strep-Tag II release from the fusion protein resulted in a band shift on the gel and weakening of the Strep-Tactin^® ^HRP immunoblot signal compared to the untreated control.

Next, we exchanged the Strep-Tag II with a larger cleavage target. The model peptide FLS (26 aa) consisted of the FLAG and Strep-Tag II octapeptides that were linked by a flexible region. After expression of *Ecotin-Ubiquitin-FLS *and osmolysis, the periplasmic fraction was treated as described above and digested for 16 h. The periplasm was also incubated in cleavage reaction buffer without UCH-L3 enzyme to exclude digestion by endogenous *E. coli *proteases. Cleavage was observed in the digestion sample, but not in the controls (Fig. 3B). Thus, release of FLS was exclusively caused by UCH-L3.

### Purification, preparative cleavage and verification of the FLS peptide

Several working groups obtained different peptides and proteins from cytoplasmatic expressed *ubiquitin *fusion proteins [25-29]. An N-terminal affinity tag (e.g. 6His) was often fused to *ubiquitin*, simplifying purification.

**Figure 4:**
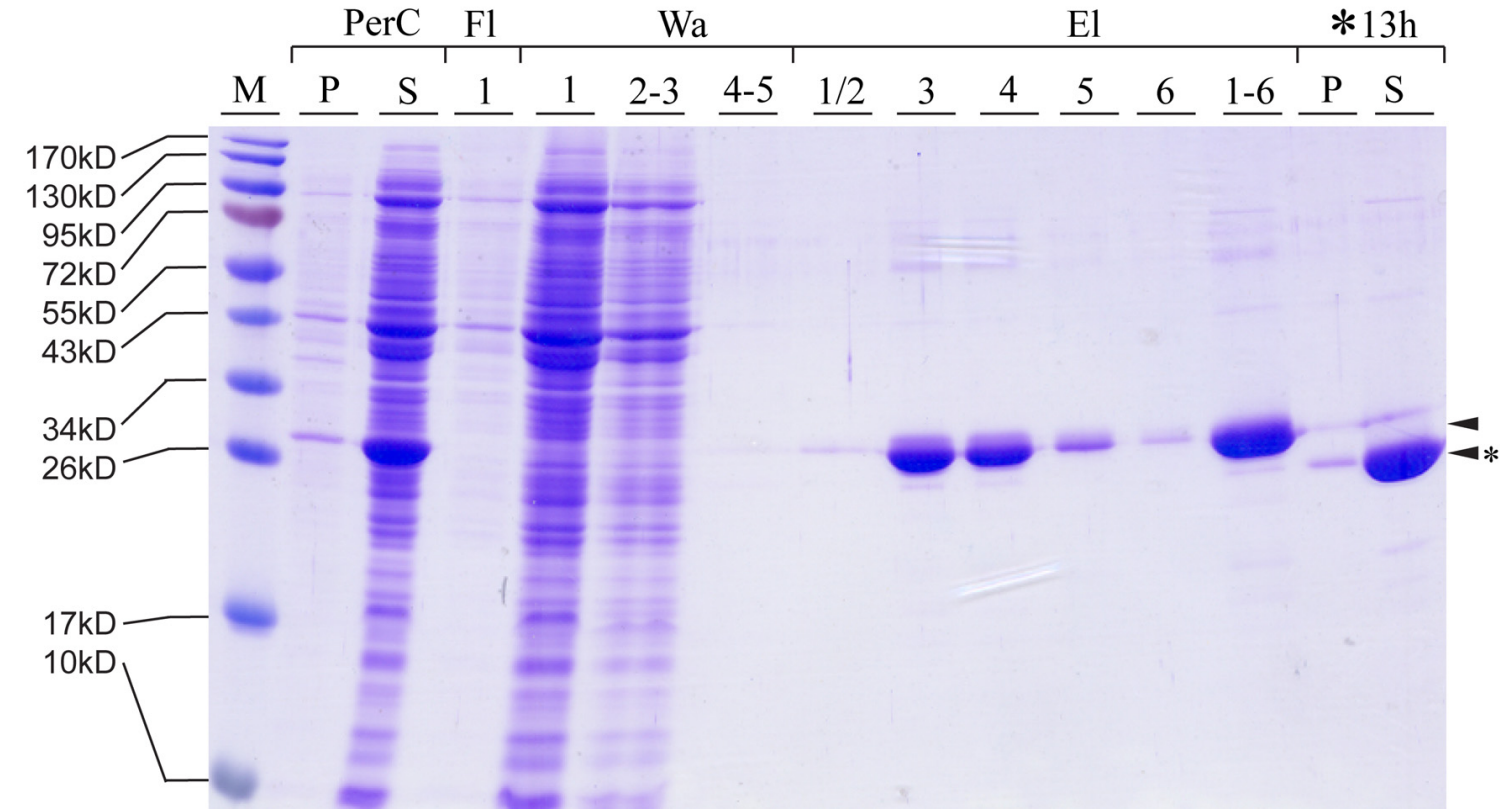
**Purification of Ecotin-Ubiquitin-FLS from periplasmic concentrate by Strep-Tactin^® ^affinity chromatography and follow-up cleavage with UCH-L3**. PerC: concentrated periplasmic solution, pellet (P) and supernatant (S). Fractions of the corresponding purification steps are numbered. Fl: flow-through, Wa: wash, El: elution, *: digestion of concentrated eluate (El 1–6) with UCH-L3 for 13 h. The upper lane represents intact recombinant protein. The cleavage product Ecotin-Ubiquitin is labeled with an asterisk.

**Figure 5:**
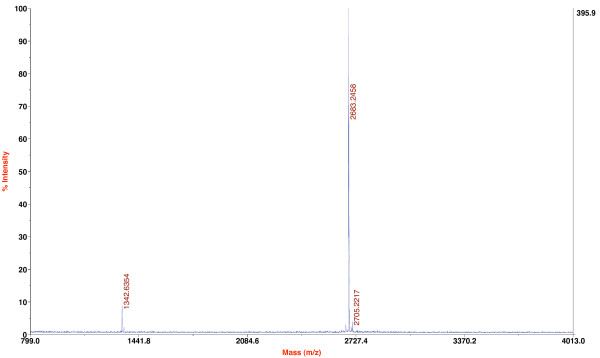
**Maldi TOF/TOF mass spectroscopic spectre for UCH-L3 digested Ecotin-Ubiquitin-FLS in the m/z range 799–4013**. The peaks at 2683 and 1342 represent the single and double protonation states of the FLS peptide, respectively.

In the current study we cloned the full-length *ecotin *gene upstream of *ubiquitin*. Although our results indicated cleavage at the C-terminus of ubiquitin by UCH-L3 it was necessary to confirm specific hydrolysis of the α-amide bond following glycine 76. Thus, we determined the molecular mass of released FLS. First, nine volumes filtrated periplasm, containing Ecotin-Ubiquitin-FLS, were mixed with one volume 10× buffer W. The solution was then concentrated by a factor of 250 and the fusion protein purified by Strep-Tactin^® ^Sepharose^® ^affinity chromatography. The eluate fractions were pooled, concentrated to 1 ml and hydrolysis carried out for 13 h with UCH-L3 (Fig. 4). The digestion probe had a purity of >95% (SDS-PAGE) with a total protein concentration of 2.24 mg/ml (recovery rate of recombinant protein, 78%). Accordingly the enzyme/substrate molar ratio was ~1:1650. Since ≥ 96% (as determined by densitometry of SDS-PAGE) of the fusion protein molecules have been cleaved by UCH-L3 the sample was directly analyzed by MALDI TOF/TOF mass spectroscopy (MS). The predicted mass of the FLS peptide was accurately determined (Fig. 5) and the peptide identity also confirmed in a collision-induced dissociation (CID) fragmentation analysis (see additional file 2: *De novo *sequencing of the putative FLS peptide by CID MS/MS).

We suggest that also larger peptides and proteins can be produced with the new technique by the use of deubiquitinating enzymes that cleave fusion proteins irrespective of their size. For instance, hydrolysis could be conducted with *S. cerevisae *ubiquitin-specific protease I [30] or the D2QL analogue [31]. The yield of target products may also be increased replacing *ecotin *with a smaller secretion moiety. However, one should consider that in fact certain ubiquitin fusion proteins are accurately cleaved *in vivo *in *E. coli *[28]. Although bacteria do not have an intrinsic ubiquitin system, various deubiquitinating proteases were found in several strains [32-34]. These endogenous ubiquitin-processing enzymes might interfere with the ubiquitin fusion system, reducing desired product yield. For general application of the system genomic deletion of these enzymes should be considered. The first amino acid of the target product should also be taken into account as alterations influence the expression rate of the fusion protein in yeast [25].

## Conclusion

We developed a novel system for the production of small peptides in *E. coli*. Different fusion proteins were generated, consisting of the periplasmic protease inhibitor *ecotin*, *mouse ubiquitin b *and either the target peptide Strep-Tag II (8 aa) or FLS (26 aa). Ecotin, encoding a 142 amino acid monomer with an implied signal peptide [35], was used to promote secretion. Ubiquitin served as substrate for rabbit ubiquitin c-terminal hydrolase-L3 to cleave off the target peptides. A further function was to enhance the fusion protein solubility. With our shake flask fermentations at 24°C we produced 18 mg periplasmic recombinant fusion protein per gram dry cell weight, corresponding to 0.75 mg Strep-Tag, respectively 1.78 mg FLS. Both peptides were efficiently removed from ubiquitin by UCH-L3 at an enzyme/substrate molar ratio of up to 1:2750. Thus, ecotin did not impede cleavage. Accurate digestion was confirmed by MALDI TOF/TOF mass spectroscopy for the FLS peptide, followed by de novo MS/MS sequencing.

## Abbreviations used

DTT: dithiothreitol; DUB: deubiquitinating enzyme; EDTA: ethylene-diamine-tetra-acetic acid; IPTG: isopropyl-β-D-thiogalactopyranoside; MTG: monothioglycerol; PCR: polymerase chain reaction; SDS: sodium dodecyl sulfate; TCA: trichloro acetic acid; UCH-L3: ubiquitin C-terminal hydrolase-L3.

## Competing interests

The authors declare that they have no competing interests.

## Authors' contributions

MP generated the fusion constructs and performed the experiments. TH optimized the osmotic shock procedure. RS and BA supervised the work and edited the manuscript.

## Supplementary Material

Additional File 1 **Ecotin-Strep expression with the pET system.** Analysis by SDS-PAGE and immunoblotting. -I and +I: before and after induction of expression, IB: inclusion bodies, Cyt: cytoplasm, Per: periplasm, CS: culture supernatant. The lower arrow with the asterisk indicates Ecotin-Strep where the signal peptide was cleaved off. Click here for file

Additional File 2 ***De novo sequencing of the putative FLS peptide by CID MS/MS***. Comparison of the y- and b- ions with an artificial FLS fragment database. Click here for file
