# Supplementary figures and images for: A novel Ecotin-Ubiquitin-Tag (ECUT) for efficient, soluble peptide production in the periplasm of Escherichia coli
Source: Microb Cell Fact. 2009 Jan 21;8:7. doi: 10.1186/1475-2859-8-7 (PMC2649888; doi:10.1186/1475-2859-8-7)

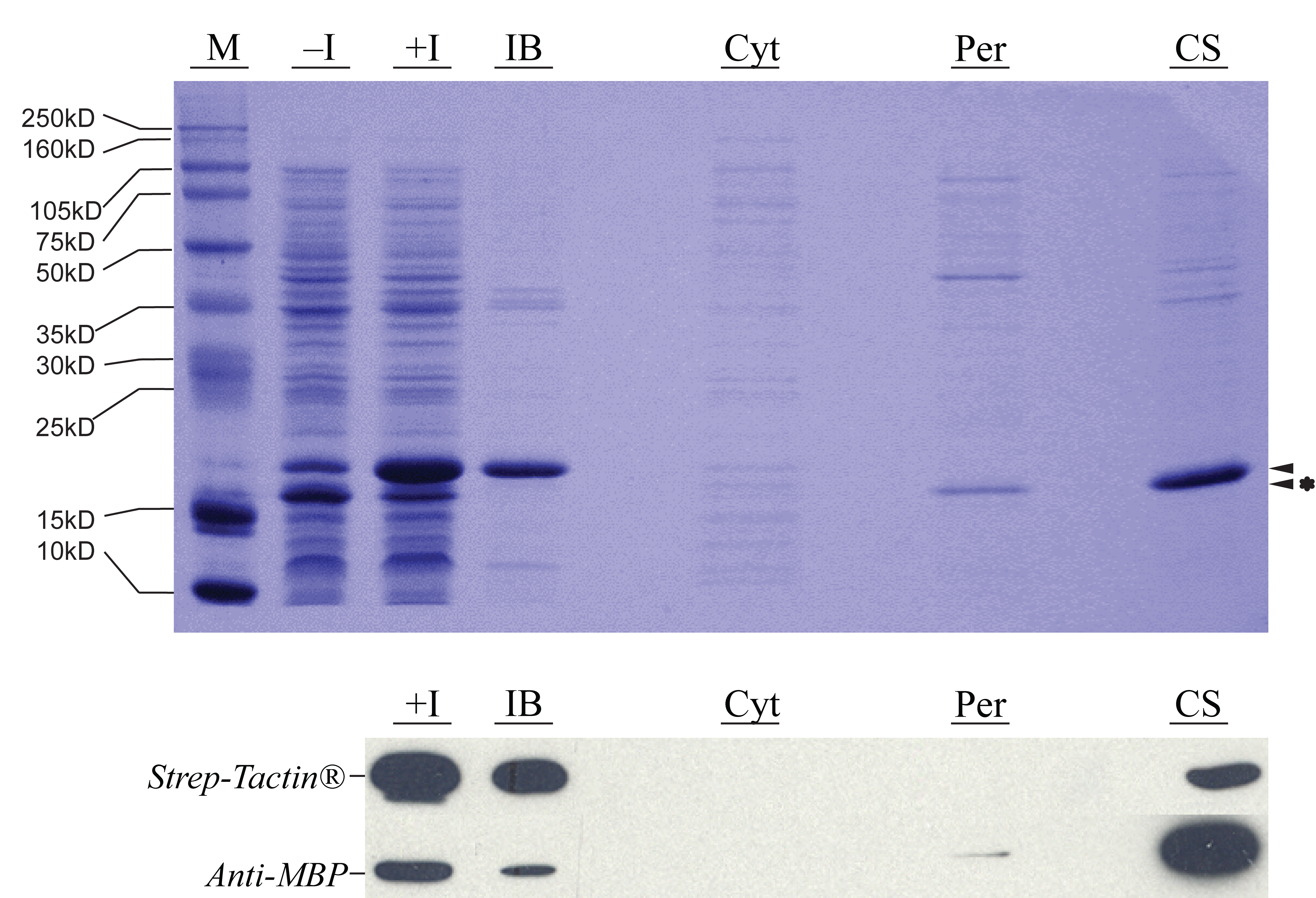

Supplement: Additional File 1 — Ecotin-Strep expression with the pET system. Analysis by SDS-PAGE and immunoblotting. -I and +I: before and after induction of expression, IB: inclusion bodies, Cyt: cytoplasm, Per: periplasm, CS: culture supernatant. The lower arrow with the asterisk indicates Ecotin-Strep where the signal peptide was cleaved off. [file 1475-2859-8-7-S1.png]

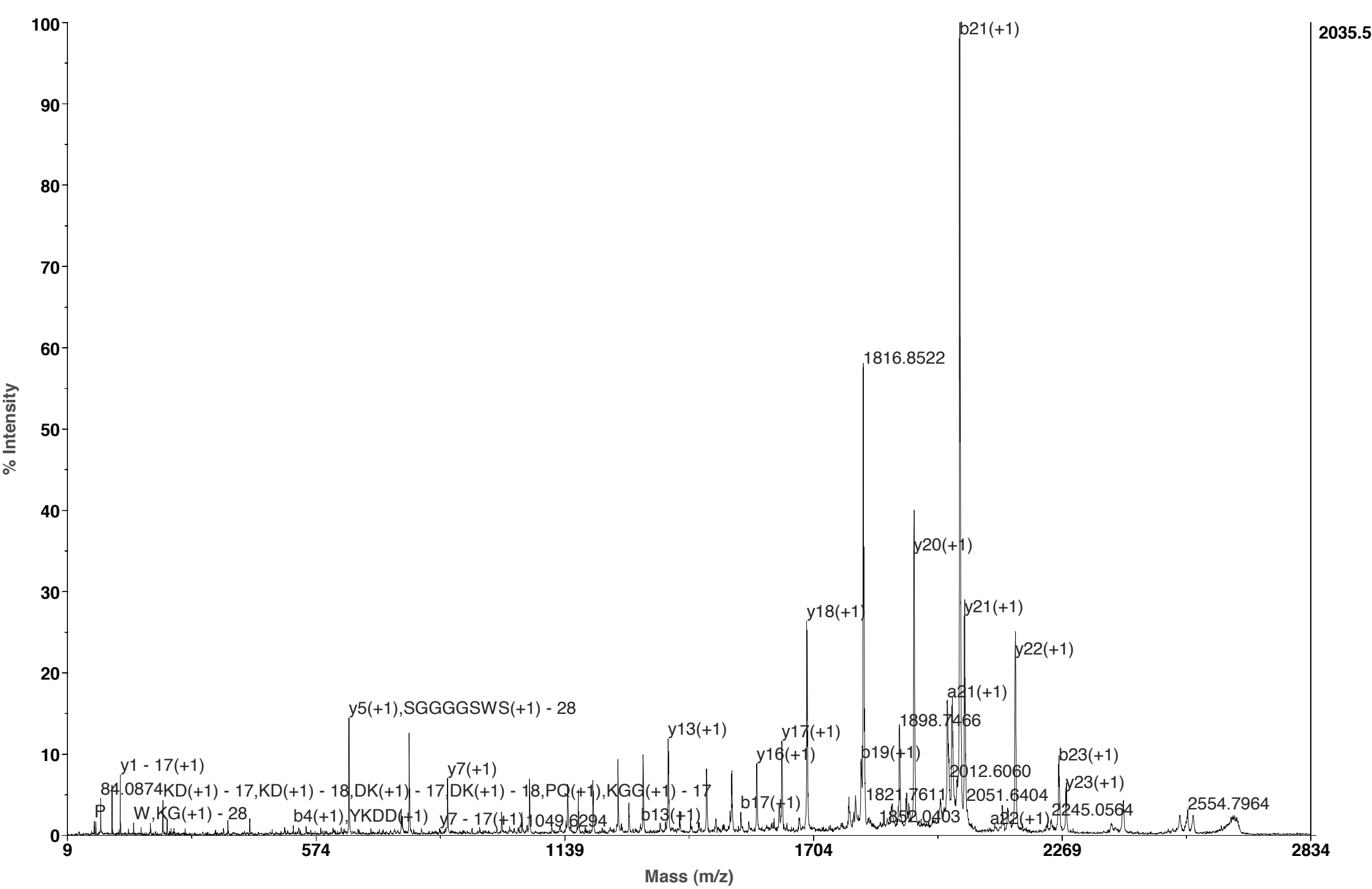

Supplement: Additional File 2 — De novo sequencing of the putative FLS peptide by CID MS/MS. Comparison of the y- and b- ions with an artificial FLS fragment database. [file 1475-2859-8-7-S2.pdf]
